# ERP Mismatch Negativity Amplitude and Asymmetry Reflect Phonological and Rapid Automatized Naming Skills in English-Speaking Kindergartners

**DOI:** 10.3389/fnhum.2021.624617

**Published:** 2021-06-18

**Authors:** Elizabeth S. Norton, Sara D. Beach, Marianna D. Eddy, Sean McWeeny, Ola Ozernov-Palchik, Nadine Gaab, John D. E. Gabrieli

**Affiliations:** ^1^Department of Communication Sciences and Disorders, Northwestern University, Evanston, IL, United States; ^2^McGovern Institute for Brain Research and Department of Brain and Cognitive Sciences, Massachusetts Institute of Technology, Cambridge, MA, United States; ^3^Department of Medical Social Sciences, Feinberg School of Medicine, and Institute for Innovations in Developmental Sciences, Northwestern University, Chicago, IL, United States; ^4^Harvard Graduate School of Education, Cambridge, MA, United States; ^5^Laboratories of Cognitive Neuroscience, Division of Developmental Medicine, Boston Children’s Hospital/Harvard Medical School, Boston, MA, United States; ^6^Institute for Medical Engineering and Science, Massachusetts Institute of Technology, Cambridge, MA, United States

**Keywords:** mismatch negativity, MMN, dyslexia, ERP, reading, phonological awareness, rapid automatized naming

## Abstract

The mismatch negativity (MMN), an electrophysiological response to an oddball auditory stimulus, is related to reading ability in many studies. There are conflicting findings regarding exactly how the MMN relates to risk or actual diagnosis of dyslexia/reading impairment, perhaps due to the heterogeneity of abilities in children with reading impairment. In this study, 166 English-speaking kindergarten children oversampled for dyslexia risk completed behavioral assessments and a speech-syllable MMN paradigm. We examined how early and late MMN mean amplitude and laterality were related to two established predictors of reading ability: phonological awareness (PA) and rapid automatized naming (RAN). In bootstrapped group analyses, late MMN amplitude was significantly greater in children with typical PA ability than low PA ability. In contrast, laterality of the early and late MMN was significantly different in children with low versus typical RAN ability. Continuous analyses controlling for child age, non-verbal IQ, and letter and word identification abilities showed the same associations between late MMN amplitude with PA and late MMN laterality with RAN. These findings suggest that amplitude of the MMN may relate to phonological representations and ability to manipulate them, whereas MMN laterality may reflect differences in brain processes that support automaticity needed for reading.

## Highlights

- Examined how reading skills relate to the mismatch negativity (MMN) in English.
- Phonological awareness (PA) and rapid automatized naming (RAN) relate to reading.
- MMN amplitude significantly related to PA.
- MMN laterality significantly related to RAN.

## Introduction

The mismatch negativity (MMN) component has been studied extensively in relation to developmental disorders of language and reading (Kujala and Näätänen, 2001; Bishop, 2007; Volkmer and Schulte-Körne, 2018). The MMN is elicited by a deviant or “oddball” stimulus within a series of standard repeated auditory stimuli (Näätänen et al., 1978, 2012). The difference in the event-related potential (ERP) response between the frequent (standard) vs. infrequent (deviant) stimuli is the MMN. The MMN is measured non-invasively at the scalp, is elicited even when the participant is not actively attending to the stimuli, and requires no behavioral response, and thus is well suited to studying infants and children in order to understand risk for later reading impairment, especially early in life.

The MMN varies in morphology and timing depending on factors such as participant age, stimulus type, stimulus timing, and reference electrode. The MMN response is thought to reflect two brain processes: an earlier pre-attentional change detection or release from stimulus-specific adaptation (Jääskeläinen et al., 2004) that is reflected in positivity over bilateral central/temporal regions, and a subsequent process that may be an involuntary attentional switch (reflected in negativity over fronto-central cortex, sometimes with slight right lateralization) (Rinne et al., 2000; Näätänen and Kreegipuu, 2011). Especially in studies of children, the MMN tends to have two separable peaks or components, an early peak around 150–250 ms, similar in latency to the adult MMN, and a later peak around 300–500 ms (Cheour et al., 1999; Morr et al., 2002). These two different peaks or components may reflect these two processes, the initial change detection and the later attentional switch. The late MMN, sometimes called the late discriminative negativity (LDN), peaks over frontal electrodes with some rightward lateralization (Rinne et al., 2000; Cheour et al., 2001; Opitz et al., 2002; Näätänen and Kreegipuu, 2011; van Zuijen et al., 2013; Todd and Fitzgerald, 2020), suggesting that it is related to processes that occur after initial processing in auditory cortex. Source models in adults estimate that there are two frontal generators of the MMN signal that are not symmetric, yielding larger MMN responses in the right hemisphere (Jemel et al., 2002), though there is debate regarding how stimulus characteristics such as timing and auditory properties relate to lateralization of the MMN response (Bishop, 2007; Näätänen et al., 2007). The late MMN tends to be stronger for speech than for tone stimuli (Korpilahti et al., 1995; Bruder et al., 2011; but see Alonso-Bua et al., 2006), possibly reflecting processes such as conscious detection of a complex stimulus change (Korpilahti et al., 2001; Bishop et al., 2011).

The relations between the MMN and reading ability, broadly construed, have been studied in a variety of languages and participants, from infants with familial risk of dyslexia to children and adults with low reading scores or diagnosed reading impairment or dyslexia (e.g., Kraus et al., 1996; Paul et al., 2006; Huttunen-Scott et al., 2008; Hommet et al., 2009; Maurer et al., 2009; Bruder et al., 2011; Neuhoff et al., 2012; Noordenbos et al., 2012; Hämäläinen et al., 2013; Plakas et al., 2013; Schaadt and Männel, 2019; for review, see Volkmer and Schulte-Körne, 2018). Further, studies have identified longitudinal associations between different MMN responses in infancy and later reading and writing abilities (Guttorm et al., 2005, 2010; Leppänen et al., 2012; van Zuijen et al., 2013; Schaadt et al., 2015). Reading impairments and familial risk for reading impairments have been variously associated with reduced amplitude, later onset, and/or differential scalp topography of the MMN, although the exact differences vary considerably across studies, with some studies finding no differences (see Bishop, 2007 for a review, Gu and Bi, 2020 for meta-analysis). The topographical asymmetry of the early and late MMN have rarely been directly considered in relation to reading ability; however, one study suggested that posterior left laterality of the MMN at kindergarten age may be a strong predictor of later reading ability (Maurer et al., 2009). One other study of infants at risk for dyslexia also found that the eventual typical reader group had a strong right-lateralized frontal mismatch response to speech syllables, whereas the dyslexia group had a left-lateralized response (van Zuijen et al., 2013). A study that grouped children by strength of right frontal MMN found that children with a negative (versus positive or neutral) right frontal MMN were less likely to have a family history of dyslexia (FHD; Guttorm et al., 2010).

The vast differences in stimuli, timing, electroencephalography (EEG) recording, and data analysis approaches may account for the different results seen across studies (McWeeny and Norton, 2020). In addition, diagnostic and grouping criteria vary even within a language or country, so one “dyslexia” group in one study may be very different from the next. (For example, some studies use the term dyslexia to refer only to an unexpected single word reading deficit, while others use the term dyslexia to apply to unexpected reading difficulties more broadly, such as with reading fluency or comprehension.) Further, within a single group of individuals with dyslexia or reading impairment, there is substantial heterogeneity in their specific reading-related abilities. Increasing evidence supports a multi-componential view of the disorder, such that there is no single underlying cause that explains all cases, but instead, multiple independent deficits alone or in combination can cause the specific reading problems characteristic of dyslexia (Pennington et al., 2012; van Bergen et al., 2014; Catts et al., 2017; Compton, 2020; O’Brien and Yeatman, 2020).

The mechanism(s) by which the MMN relates to word and text reading accuracy and speed are also not yet clear. Several hypotheses about the causes of altered MMN in groups with dyslexia or reading impairment have tended to focus on lower-level processes and environmental factors. Impairment in lower-level auditory perceptual abilities has been suggested as one cause of reduced MMN and can be observed in some children with dyslexia (Giraud and Ramus, 2013). In some studies, participants’ ability to behaviorally discriminate the auditory stimuli used in the MMN task differed or was significantly related to their MMN responses (Kraus et al., 1996; Sharma et al., 2006), but perceptual ability was found to be unrelated to the MMN response in other studies (Paul et al., 2006; Stoodley et al., 2006; Bruder et al., 2011). Another potential explanation for MMN differences in dyslexia is reduced language experience; however, infants with a FHD show attenuated MMNs within hours of birth (Leppänen et al., 1999, 2002, 2010; van Leeuwen et al., 2008; van Zuijen et al., 2013). Thus, the relation between MMN and reading seems to not be fully explained by deficits in auditory processing or discrimination ability or by postnatal language experience. Further, a meta-analysis of MMN studies found that there was no overall significant difference in MMN response amplitudes to non-speech stimuli in dyslexia, whereas there was a significantly reduced MMN in dyslexia in studies that used speech stimuli (Gu and Bi, 2020).

Given that MMN responses to speech are more strongly linked with dyslexia, an alternative hypothesis is that the MMN relates to reading ability because of shared neural processes relating to efficient auditory, linguistic, and phonological processing. Deficits in phonological awareness (PA), the ability to identify and manipulate language sounds, are common in dyslexia (Bradley and Bryant, 1978; Morris et al., 1998; Pennington et al., 2012). A clearer understanding of how phonological abilities relate to the MMN amplitude and asymmetry may provide greater information on the nature of the relationship between MMN and reading more broadly. Some evidence suggests that a lack of precise timing of neural oscillations or firing patterns that are relevant to processing speech at the syllable and phoneme level could account for the phonological and letter-sound mapping deficits that are common in dyslexia (Lehongre et al., 2011, 2013; Goswami, 2015; Hancock et al., 2017). Auditory timing and attention-shifting deficits have also been suggested as underlying the relation between MMN and reading deficits (Meyer and Schaadt, 2020). Other accounts suggest that individuals with dyslexia have poor PA because of diminished access to phonological representations, with the representations themselves intact (Boets et al., 2013).

Access to linguistic representations and sound-symbol mappings are also central to rapid automatized naming (RAN), a task measuring the speed with which one can retrieve and produce the names of a series of highly familiar printed items such as colors or letters. RAN is implicated in dyslexia (Wolf and Bowers, 1999) and thought to reflect the automaticity of multiple processes that are shared with reading (Norton and Wolf, 2012). Both PA and RAN can be assessed before children learn to read and predict later reading ability across languages (Schatschneider et al., 2004; Landerl et al., 2013). PA and RAN abilities are somewhat correlated, but each one accounts for unique variance in reading ability (Manis et al., 2000) and data suggest that they have distinct genetic correlates (Petrill et al., 2006; Naples et al., 2009). Importantly, children with deficits in PA versus RAN have different patterns of neural activation and connectivity during reading (Norton et al., 2014).

The few studies that have examined how the MMN relates to PA and RAN used somewhat small samples and yielded inconsistent results. One study observed that in a group of 37 Finnish 5- and 6-year-olds, early MMN amplitude was related to PA, whereas late MMN amplitude correlated with RAN (Hämäläinen et al., 2015). Among Finnish infants (*n* = 47), early MMN amplitude correlated with future RAN abilities at age 5.5 and infants’ late MMN correlated with their future PA at age 3.5 (Leppänen et al., 2010). However, in *n* = 38 Dutch preschoolers, the MMN correlated with future performance on speeded reading measures, but not PA (Plakas et al., 2013). Another study of beginning readers age 6–7 in Germany grouped children into good (*n* = 15) versus poor (*n* = 19) performers on a phonological measure that asked them to decide if a pair of “similar or similar sounding words” was identical; the poor performance group had reduced late MMN amplitude (Bitz et al., 2007).

Studying these specific skills that have a strong relation with reading may provide greater mechanistic insight because they are more focused and dimensional than the general diagnostic category of dyslexia or grouping based on word reading scores. Researchers and leaders in the area of mental health have advanced a dimensional, symptoms-based approach (National Institute of Mental Health’s Research Domain Criteria, or RDoC; Cuthbert and Insel, 2013) in order to parse heterogeneity in diagnostic categories, which are particularly varied in development (Mittal and Wakschlag, 2017; Damme et al., 2020) and ultimately, to better understand the neurobiology of disorders. Thus, in this study, we explicitly tested how MMN measures related to PA and RAN, two crucial reading-related skills and deficits in dyslexia. Studying these specific skills may allow us to better characterize how the MMN relates to reading. We used a large community sample of kindergarten-age children with varied pre-reading abilities, oversampled for risk for dyslexia. Kindergarten is an optimal age to study this phenomenon because this is the age when PA and RAN can first be measured reliably, and when early intervention for reading may be most effective (Wanzek and Vaughn, 2007). We chose *a priori* to examine the amplitude and latency of the early and late MMN components, using electrodes and time windows from the literature or based on the whole-sample waveform, in order to reduce experimenter degrees of freedom and promote rigorous, reproducible practices (Kappenman and Luck, 2016).

## Materials and Methods

### Participants

Participants were enrolled in The READ Study (**R**esearch on the **E**arly **A**ttributes of **D**yslexia), a larger study of reading development and risk for dyslexia at MIT and Boston Children’s Hospital, which included behavioral assessment as well as EEG and (f)MRI. Participants were recruited from 20 schools in eastern Massachusetts and Rhode Island, which included public (district and charter), private, and religious schools in urban and suburban areas. With parental consent, kindergarten-age children completed a short behavioral screening in their schools. A subset of the children screened in schools, oversampled for low scores (see section “Behavioral Assessment and Risk Status”), was then invited to participate in the full study which included MRI and EEG. The study was approved by institutional review boards at MIT and Boston Children’s Hospital. Parents gave written consent and children gave verbal assent to participate. Families received bookstore gift cards and reimbursement for travel costs as thanks for participating in the EEG session.

All participants met eligibility criteria including: born after at least 35.5 weeks gestation; consistent exposure to English beginning before age 12 months from a native English speaker; no sensory or perceptual difficulties other than corrected vision; no history of head or brain injury or trauma; no current medications affecting the nervous system; no diagnosis of major neurodevelopmental or psychiatric disorders (e.g., autism spectrum disorder, depression). Children who were at risk for dyslexia were oversampled; a greater proportion of children with low scores (lowest quartile of the sample, see details below) on measures of RAN, PA, and/or letter knowledge were invited to participate in the full study.

The present study sample included 166 children who participated in behavioral assessment and EEG during either the spring/summer before kindergarten or the fall of kindergarten. The sample included 84 boys and 82 girls aged 4 years 10 months to 6 years 8 months (mean = 5 years 6 months). An additional eight children completed the EEG session but were excluded from analysis due to excessive body movement or artifacts resulting in too few usable trials, or poor EEG data quality due to very thick or braided hair.

Parents completed a questionnaire about their child’s developmental history and demographic information. The race and ethnicity of children in the sample was: 2 American Indian or Alaska Native, 1 Asian/Asian-American, 30 Black/African-American, 114 white, and 10 more than one race; 19 children were Hispanic or Latinx (an additional 9 families did not report the child’s race/ethnicity). The mother’s education, an indicator of SES, was completion of high school for 12 participants, some college or 2-year degree for 32 participants, and college degree or higher for 117 participants (not data for 1 participant). Parents also reported on their child’s handedness; 140 children were right-handed, 6 were ambidextrous, and 18 were left-handed (no data for 2 participants). Parents also completed a questionnaire about their family’s history of dyslexia; 36 children in the current sample (22%) had a parent or sibling diagnosed with dyslexia.

### Behavioral Assessment and Risk Status

Trained research assistants (most of whom were master’s-level speech-language pathology students) administered a comprehensive behavioral assessment battery. All assessment sessions were audio recorded and administration and scoring were checked for accuracy and tester reliability. For each measure, standard scores based on age were determined. The tests did not include score norms for 4-year-olds, so score norms for age 5;0 (years; months) were used for the children in the sample who were age 4;10–4;11.

For this analysis, we focused on only two pre-reading skills, PA and RAN. PA was assessed using the Elision and Blending Words subtests from the Comprehensive Test of Phonological Processing (CTOPP; Wagner et al., 1999), which has a scaled score mean of 10 and SD of 3. In the Elision subtest, the child is given a word and asked to remove a syllable or phoneme, and then provide the remaining sounds. For example, if the item was “say play without saying/p/,” the correct answer would be “lay.” For the Blending Words subtest, the child hears a series of isolated phonemes and is asked to blend the sounds together to make a word. For example, for the prompt “what word do these sounds make: /b//e//k/,” where the correct answer would be “back.” These subtests were selected because they assess basic aspects of PA and were collected for all participants. A composite score was obtained by calculating the mean scaled score of the two subtests. In the case that a child had valid data for only one subtest or failed the practice items on one subtest (*n* = 12), the scaled score from the one valid test was used. RAN was assessed using the Objects, Colors, and Letters subtests of the RAN-Rapid Alternating Stimulus Tests (RAN-RAS Tests; Wolf and Denckla, 2005). This test has a mean standard score of 100 and SD of 10. For the RAN-RAS tests, children are asked to name the items in the array as quickly as possible without making mistakes, naming across each row from top to bottom. There is first a practice set of 2 rows of 5 items each; children who could not identify each of the five letter stimuli during practice (*n* = 22) did not complete the Letters subtest and their scores from only Objects and Colors were included in their composite. A mean of valid scores was calculated to create a composite RAN score.

In addition, non-verbal cognitive ability, letter knowledge, and word reading were assessed. Non-verbal cognitive ability was assessed via the Matrices subtest of the Kaufman Brief Intelligence Test, Second Edition (KBIT-2; Kaufman and Kaufman, 2004); all children scored in the typical range (age-based standard score ≥ 80). Children also completed the Letter ID and Word ID subtests from the Woodcock Reading Mastery Tests, Revised/Normative Update (WRMT-R/NU; Woodcock, 1998) as measures of untimed letter name knowledge and single-word reading, respectively; many children could not yet read any words.

Dyslexia risk was oversampled by inviting a larger proportion of children with PA, RAN, or letter knowledge scores in the lower 25% of the sample to participate in the brain imaging portion of the study (as well as longitudinal follow-up, not reported here). Risk was categorized as scoring in the bottom 25% for age in the sample on at least one composite measure of one of three constructs of interest: PA, RAN, or letter ID (constructs based on Schatschneider et al., 2004); similar prospective risk studies use a 25% criterion for risk (e.g., Schaadt et al., 2015). Letter knowledge was not considered as a risk criterion in the present analysis for two reasons; first, letter knowledge does not have a strong proposed theoretical link to the MMN, whereas auditory and automatic processing do, and second, letter knowledge is highly influenced by previous school and home literacy experiences related to socio-economic status (Duncan and Seymour, 2000; Robins et al., 2014) and is more transient predictor of reading until nearly all children reach ceiling at the end of kindergarten (Paris, 2005).

### Group Assignment

For the group analyses, participants were grouped by low versus typical ability for PA and for RAN. There are no universal tests or cutoffs that determine categorially poor performance on PA and RAN measures. This dichotomous approach was taken so that results could be considered similar to a “pass” or “fail” on a screening measure. We calculated the 25th percentile for each construct based on a larger sample for this study of over 1,154 children (inclusion criteria: had data for these measures, proficient English speaker, KBIT non-verbal IQ standard score > 79, and age < 79 months; see Ozernov-Palchik et al., 2017 for full details of the larger sample). Thus, cutoffs were a mean standard score of 89.33 or below for RAN (*n* = 55) and mean scaled score of 8.0 or below for PA (*n* = 40). Thus, the “low” groups should not differ on their degree of impairment relative to the typical groups. Thirteen children met criteria for both Low PA and Low RAN and thus were included in both groups. There were no significant differences between the Low PA vs. Typical PA or Low RAN vs. Typical RAN groups in terms of age, biological sex, or handedness (independent samples *t*-test or chi square, all *p* > 0.05). The Low RAN group did not differ in terms of non-verbal IQ or word ID standard scores from their peers in the Typical RAN group, but the Low PA group had significantly lower non-verbal IQ (*p* < 0.001) and word ID standard scores (*p* = 0.008) as compared to the Typical PA group, though the group means were well within the average range. Because children were assessed in kindergarten, reading 0 words correctly can still yield a standard score in the typical range. In order to further describe the sample, we examined how many children included here met similar criteria for scoring in the bottom 25% for a composite letter knowledge (letter name and letter sound) measure. Twenty-five children met criteria for risk based on letter knowledge; of these, 18 met risk criteria for PA and/or RAN as well. Overall, when FHD and the three risk constructs were considered in total, 65 children in this sample had no risk factors identified and 101 children had at least one risk factor identified.

### Stimuli and Procedure

We used an oddball MMN paradigm with natural speech syllables /ba/ and /da/ as stimuli (as in Lachmann et al., 2005; Alonso-Bua et al., 2006; Neuhoff et al., 2012). Stimuli were recorded from an adult female native English speaker. Stimuli lasted approximately 200 ms (/da/199 ms, /ba/201 ms) and were equated for root mean square loudness using Praat software (Boersma and Weenink, 2010). Syllable stimuli were presented with a 500 ms stimulus onset asynchrony (e.g., Paul et al., 2006) using StimPres software (NeuroCognition Laboratory). This short SOA was chosen because group differences in individuals with versus without dyslexia have been reported more often for short rather than longer SOAs (Bishop, 2007), and it allowed for presentation of many trials in a shorter recording time that would be well-tolerated by children.

In order to ensure that our MMN measure reflected general auditory processing and was not due to a particular characteristic of the standard or deviant stimulus, participants completed two runs of the experiment: one with /ba/ as the standard and one with /da/ as the standard, and the other stimulus the deviant. The order of the two runs was counterbalanced across participants and participants took a short break between runs. Data for the two standards and the two deviants were collapsed for analysis. This approach of using each stimulus as both the standard and the deviant and collapsing across all standards vs. deviants is recommended by multiple reviews as an approach to minimize confounds of particular auditory stimuli and instead most accurately reflect the underlying change detection mechanism that is of interest in the MMN (Bishop, 2007; Schwartz et al., 2018). We visually confirmed that the overall morphology of the waves was similar. The paradigm included a total of 2,400 total trials (1,200 trials per run). In each run, 10% of trials were deviants; each deviant trial was preceded by at least three consecutive standard trials. Before EEG recording began for each of the runs, 20 s of “practice” stimuli were played in order to familiarize children with the stimuli and ensure that they could hear the stimuli well.

### Electroencephalography Recording

During EEG recording, children sat in a comfortable armchair in an acoustically- and electrically shielded booth. The auditory stimuli were played via earphones fitted with child-size in-ear foam tips (ER-1 earphones with ER-14B tips, Etymôtic Research Inc., Elk Grove Village, IL, United States). Children watched a video of their choice on a monitor with the video’s sound muted. Researchers and parents were seated outside the booth and observed the child via a video monitor.

EEG was recorded using the Biosemi ActiveTwo System (Biosemi B.V., Amsterdam). Recordings were made in single-ended mode that amplifies the difference between each electrode site and a common mode sensor (CMS) electrode with referencing off-line. The impedance does not need to be lowered with this system due to the combination of pre-amplifiers at each electrode site, a driven right leg (DRL) circuit, and high electrical isolation (see Kappenman and Luck, 2010). Offset values for each electrode were kept below 40 mV.

Active Ag-AgCl electrodes were affixed to an elastic fabric cap appropriate for the child’s head size (Electro-Cap Inc., Eaton, OH). EEG was recorded from 64 scalp sites arranged in 10–20 system positioning (electrodes at locations Fp 1/z/2; AF 7/3/z/4/8; F 7/5/3/1/z/2/4/6/8, FC 7/5/3/1/z/2/4/6/8, C 5/3/1/z/2/4/6, T7/8, CP 5/3/1/z/2/4/6, TP 7/8, P 9/7/5/3/1/z/2/4/6/8/10, PO 7/3/z/4/8, O 1/z/2 and Iz). Electrodes were also affixed to the right and left mastoids; data were referenced to these electrodes offline. Electro-oculogram was recorded from the lateral canthus of the right eye and the infraorbital ridge of the left eye. EEG was recorded with a low-pass hardware filter with a half-power cutoff at 104 Hz and digitized at 512 Hz with 24 bits of resolution.

### Electroencephalography/Event-Related Potential Analysis

#### Preprocessing and Artifact Rejection

Analyses were conducted using EEGLab v.17 (Delorme and Makeig, 2004)^1^ and ERPLab v.7 (Lopez-Calderon and Luck, 2014)^2^ software packages running in Matlab R2017b (MathWorks Inc., Natick, MA, United States). Data from occipital electrodes P9, P10, and Iz were excluded from import and further analysis due to frequent artifacts. Data were imported, referenced to the average of the mastoid electrodes, high-pass filtered at 0.1 Hz (half-power cutoff), then epoched with baseline correction with a 100 ms pre-stimulus baseline period and 550 ms post-stimulus onset period.

Trials with artifact (including eye blinks or movements and head/body motion) were rejected prior to averaging. ERPLab’s artifact rejection functions were used to identify and exclude trials with a step-like artifact (deviation of at least 75 μv over any 200 ms window, measured in 50ms increments over the entire epoch of −100 to 550 ms) or a moving window artifact (deviation of at least 100 μv over any 400 ms window, measured in 100 ms increments over the epoch). Accuracy of artifact detection was visually confirmed for each subject; thresholds were adjusted for individual subjects in order to obtain the most accurate rejection of artifacts (Luck, 2014). Retaining the average mastoid reference for visual inspection and artifact rejection allowed us to identify channels where there was poor quality data for a short interval that needed to be rejected or when there was consistently poor quality and the channel needed to be interpolated. At most, two channels were interpolated. The participants included in these analyses had at least 50 usable deviant trials remaining after artifact rejection (as in Bruder et al., 2011).

After artifact rejection, remaining trials were re-referenced to an average reference of all scalp electrodes (e.g., Stoodley et al., 2006). Epochs of each trial type (standard, deviant) were then averaged together. The MMN difference wave was calculated for each individual as the waveform for deviant trials minus an equal number of standard trials. Even though we used mean amplitude measures that are less subject to noise, this approach for MMN studies in general should minimize effects of noise on peak measurements (Luck, 2014). Measurements were then performed for each individual. Finally, for visualizations, data were low-pass filtered at 40 Hz (half-power cutoff).

#### Mismatch Negativity Measurements and Analysis

Our analyses focused on the regions of the most pronounced MMN response observed in previous studies of the speech-sound MMN in children, including central (C) electrodes for the early MMN and frontal (F) electrodes for the late MMN (e.g., Lachmann et al., 2005; Bruder et al., 2011). The mean of three adjacent electrodes over each hemisphere in each region (left frontal F1/3/5, right frontal F2/4/6, left central 1/3/5, right central 2/4/6) was taken to minimize the effects of noise in individual channels. These frontal and central regions were chosen as they are commonly examined in other studies. Our analysis in relation to behavioral measures approach focuses on the regions with the strongest response in order to minimize the chance of spurious findings that can occur by looking at a large number of electrodes without correction for multiple comparisons, or the reduced power that occurs when a broad approach with many comparisons is undertaken (e.g., Keil et al., 2014; Luck and Gaspelin, 2017). Many previous papers examining the MMN and reading record from a large number of electrodes yet focus in on a set of pre-determined electrodes that best reflect the MMN (e.g., Schulte-Körne et al., 1998, 2001; Alonso-Bua et al., 2006; van Zuijen et al., 2013).

**FIGURE 1:**
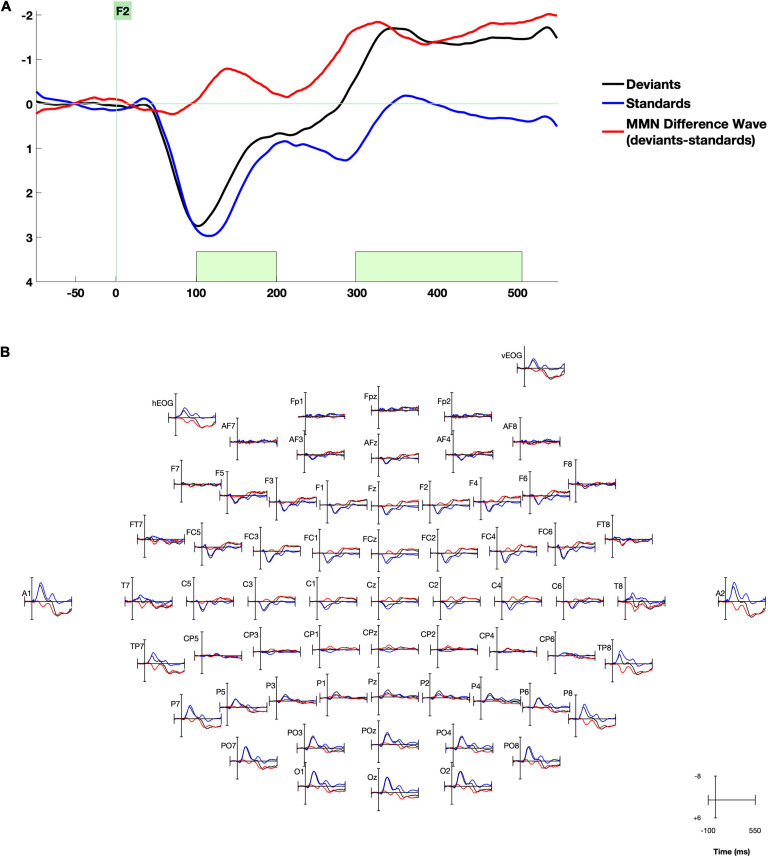
Whole-group grand average waveforms. **(A)** Data are presented at right frontal electrode site F2. Time windows selected for analysis are highlighted in green. **(B)** Data from all electrodes processed. Negative is plotted up.

Time windows of interest were determined from the MMN peaks in the whole-group grand average response (see example waveform in Figure 1), as the timing of the MMN depends heavily on the stimuli used and the presentation rate. Because our research question was focused on correlations between MMN responses and behavior, this approach of selecting the MMN location based on previous literature and time window based on whole-group grand average (Kappenman and Luck, 2016) prevents bias in our analysis. The mean amplitude of the MMN difference wave was measured in the early and late time windows using the ERPLab measurement tool. Before proceeding with analysis, it was confirmed that there were no outliers > 3 SD from the mean on any behavioral or ERP measure.

Laterality of the MMN was calculated for the early and late time windows using the equation: (−left + right)/((left^2^ + right^2^)^1/2^) (as in previous MMN work in children, e.g., Ikezawa et al., 2008), where left and right are the mean values of the three electrodes in each cluster. More positive laterality values indicate a greater (more negative) MMN amplitude on the right.

#### Analytic Strategy

Groups’ MMN measures (mean amplitude and laterality measures in early and late time windows) were compared using bootstrapped two-tailed independent-samples *t*-tests, with 10,000 samples at a 95% confidence interval level. The bootstrapping approach was used because the MMN data were not normally distributed and keeping them in their original units rather than transforming the data to meet assumptions was preferable for ease of interpretation and comparison with other literature. Similarly, partial correlation analyses were bootstrapped with 10,000 samples at a 95% confidence interval level. Partial correlations controlled for age in months, non-verbal IQ raw score, and Word ID raw score (raw scores were used because age was also included in the model); in the analysis of the PA relative to ERP measures RAN was controlled, and vice versa. Analyses were conducted in IBM SPSS Statistics 26.

## Results

### Behavioral Scores

Standard scores for the behavioral assessments are reported in Table 1. Forty-one percent of the children in the sample were pre-readers (correctly read at most one word on WRMT-R Word ID). RAN and PA Composite standard scores were weakly and not significantly correlated (*r* = 0.114, *p* = 0.144), which may be due to the sampling strategy of enrolling higher proportions of children who had low scores in either of these areas; further, these scores may be correlated more strongly in older children who have greater automaticity in phonological processing.

**TABLE 1:** Scores on behavioral measures for the full sample.

Measure	Mean	SD	Range
KBIT-2 Matrices Standard Score	99.3	9.8	80–131
PA Composite Standard Score	10.0	2.2	6–16
RAN Composite Standard Score	96.4	14.7	59–122.33
WRMT-R Word ID Raw Score	8.1	13.1	0–68

### Group Grand Average MMN Response

After artifact rejection, a mean of 130 deviant trials were included per participant (minimum 50, maximum 205). Group grand average waveforms for standard and deviant stimuli and the MMN difference wave are displayed in Figure 1. The canonical pattern of bilateral central/temporal positivity and central negativity around 100–200 ms and fronto-central negativity around 300–500 ms were present (Bruder et al., 2011; Leppänen et al., 2012). We assessed the significance of the early and late MMN by testing whether the mean values of the standard-deviant difference wave in each hemisphere in each time window/region of interest (left and right central electrode groups in the early MMN time window, and left and right frontal electrode groups in the late time window) was significantly different than zero, with one-sample *t*-tests bootstrapped with 5,000 samples. All four measures were significantly different from zero (all bootstrapped *p*s < 0.001, Hedge’s effect size range −0.261 to −0.636).

### Group Comparisons

**FIGURE 2:**
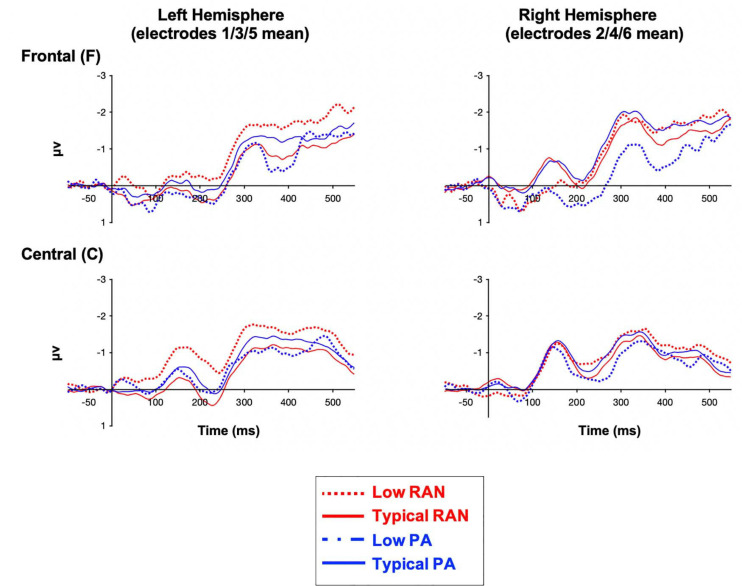
Grand average ERP MMN waveforms (deviant-standard difference wave) by group and region of interest. Negative is plotted up.

**FIGURE 3:**
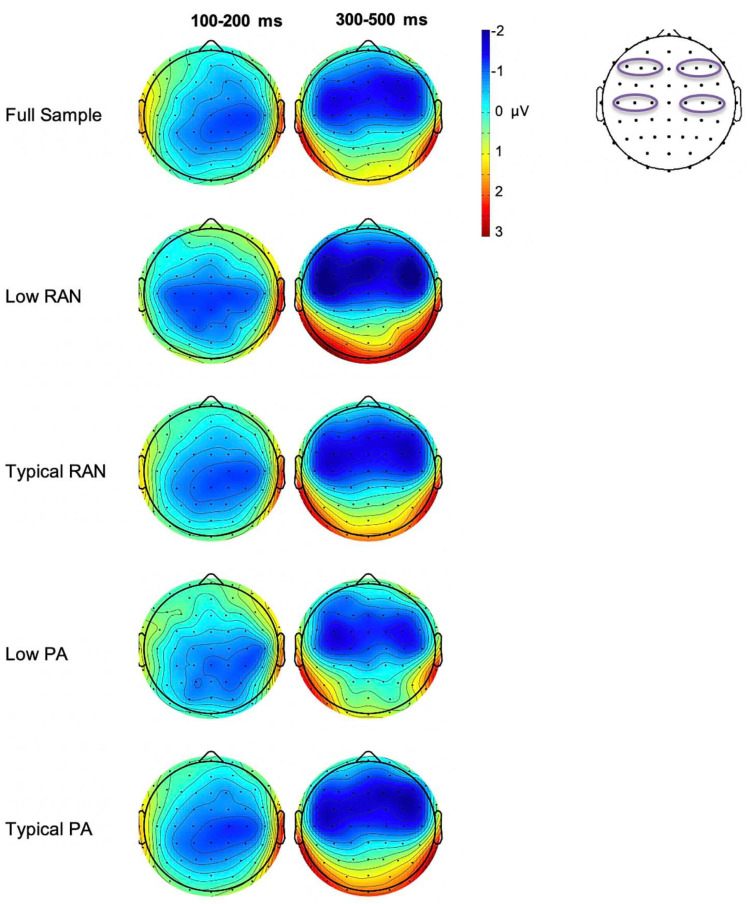
Whole group and subgroup average scalp voltage maps of the MMN difference wave mean amplitude for early (100–200 ms) and late (300–500 ms) time windows. Electrodes used in analysis are outlined on the scalp diagram on the right. Note that there is a large overlap in the participants in the Typical PA and RAN groups (*n* = 84 that are included in both), which yields similar plots.

The Low vs. Typical groups for both PA and RAN did not differ in the number of usable deviant trials included (*p* > 0.72). The measurements for all groups are provided in Table 2. The MMN difference waves for the sites included in analysis are plotted by group in Figure 2. Scalp maps of the mean amplitude of the MMN in the early and late time windows are also presented for each group in Figure 3.

**TABLE 2:** Early and late MMN measurements and group comparisons for Low and Typical RAN and PA groups.

		RAN groups’ measures (*M* ± SD)		Bootstrapped statistics		
		
		Low RAN (*n* = 55)	Typical RAN (*n* = 111)	*p*-value	95% CI of difference	Hedges’ *g*
Early central MMN (100–200 ms)	Right central amplitude	−0.844 ± 1.422	−0.905 ± 1.383	0.793	−0.409, 0.508	0.04
	Laterality index	0.102 ± 0.838	−0.385 ± 0.855	<0.001***	0.212, 0.756	0.57
Late frontal MMN (300–500 ms)	Right frontal amplitude	−1.701 ± 2.411	−1.448 ± 2.399	0.528	−1.024, 0.513	0.11
	Laterality index	−0.058 ± 0.706	0.207 ± 0.739	0.026*	−0.494, −0.029	0.36

		**PA groups’ measures (M ± SD)**		**Bootstrapped statistics**		
		
		**Low PA (*n* = 40)**	**Typical PA (*n* = 126)**	***p*-value**	**95% CI of difference**	**Hedges’ *g***

Early central MMN (100–200 ms)	Right central amplitude	−0.750 ± 1.310	−0.927 ± 1.420	0.466	−0.292, 0.654	0.13
	Laterality index	−0.222 ± 0.834	−0.223 ± 0.894	0.992	−0.301, 0.304	0.00
Late frontal MMN (300–500 ms)	Right frontal amplitude	−0.879 ± 2.430	−1.739 ± 2.361	0.044*	0.028, 1.699	0.36
	Laterality index	0.002 ± 0.790	0.156 ± 0.718	0.272	−0.429, 0.115	0.21

Group comparisons are based on independent-samples t-tests, two-tailed, bootstrapped with 10,000 samples. *p < 0.05, ***p < 0.001. Mean amplitude is measured in in μV. A more positive laterality index indicates a more rightward laterality of the negative MMN.

#### Rapid Automatized Naming

Typical (better) RAN ability was associated with significantly more leftward laterality in the early MMN measured over central electrodes, and more rightward laterality of the late frontal MMN, with medium to small effect sizes of 0.57 and 0.36, respectively (Hedges’ *g*, same interpretation of effect size as Cohen’s *d*, Cohen, 1988).

#### Phonological Awareness

Typical (better) PA was associated with significantly more negative mean amplitudes in the right frontal region for the late MMN, with an effect size of *g* = 0.36. There were no significant associations observed between PA groups and laterality of the MMN components.

### Correlational Analyses

In order to assess whether the patterns observed in the group analysis were consistent in a continuous analysis and to account for potential confounding variables, we ran partial correlations between the composite standard scores for RAN and PA with the MMN measures, controlling for child age in months, non-verbal IQ raw score, and Word ID raw score; further, RAN correlations control for PA, and PA correlations control for RAN. These correlations are provided in Table 3. The same overall pattern of results was present, except that the early MMN laterality association was no longer significant (*p* = 0.064).

**TABLE 3:** Partial correlations among PA and RAN measures and MMN measures.

	Early MMN right central amplitude	Early MMN laterality	Late MMN right frontal amplitude	Late MMN laterality
RAN mean standard score	0.002	−0.143	−0.027	0.171*
95% CI	[−0.173, 0.162]	[−0.286, 0.005]	[−0.173, 0.114]	[0.022, 0.319]
PA mean standard score	−0.117	−0.045	−0.204**	0.040
95% CI	[−0.264, 0.033]	[−0.196, 0.111]	[−0.357, −0.040]	[−0.125, 0.198]

Two-tailed, bootstrapped partial Pearson correlations with 10,000 samples.

*p< 0.05, **p < 0.01.

## Discussion

In this study, we observed that the ERP MMN mean amplitude and laterality were related to two important predictors of reading and deficits in dyslexia: PA and RAN. This is the first study to focus on parsing out relations between measures of the MMN and these crucial reading-related skills, as well as the first to specifically examine MMN laterality differences associated with these abilities. It is also the largest study of the MMN in kindergarten children, to our knowledge. Results revealed that laterality of both the early and late MMN components was significantly different in children with low vs. typical RAN, and amplitude of the late MMN in right frontal regions was greater for children with typical than low PA. In addition to categorical/group analyses, partial correlation analyses with continuous measures of PA and RAN (accounting for child age, non-verbal IQ, word reading ability, and the other construct, e.g., PA analysis controlling for RAN) showed similar patterns of associations, though the association between the early MMN laterality and RAN did not reach significance.

The MMN response (the difference between deviant and standard stimuli) that we observed to speech syllable stimuli /ba/ and /da/ syllables was generally similar to that seen in previous work, despite the variations in stimuli characteristics and presentation across previous studies. Here, the MMN was characterized by an early negative wave with a peak at about 150 ms with the largest amplitude visible over central sites and a positivity at bilateral temporal sites. The late MMN was characterized by a wide, plateau-like wave beginning just after 300 ms and extending through 500 ms that appeared most negative over frontal sites with slight right lateralization. The characteristics of the whole-group average MMN waveform observed in this study are consistent with previous MMN studies that also used syllable stimuli (Hommet et al., 2009; Neuhoff et al., 2012), even though those studies examined older children and adults whose native language was French or German. Right-lateralized frontal early MMN responses to speech-syllable stimuli were also observed in previous studies of Spanish children age 4–8 (Alonso-Bua et al., 2006).

### Mismatch Negativity Amplitude in Relation to Reading-Related Abilities

The goal of this study was to examine how MMN amplitude and laterality measures related to RAN and PA, which are widely considered to be two of the most robust predictors of later reading skills. Our findings are broadly consistent with previous studies that observed that stronger MMN amplitudes were related to better scores on various measures of reading performance. One of the most common associations in previous studies has been between the mean amplitude of the late MMN and reading ability, primarily in older children (Schulte-Körne et al., 1999; van Leeuwen et al., 2008; Halliday et al., 2014). Here, we observed significantly greater late MMN amplitude in the Typical PA group than in the Low PA group. PA is one of the most common deficits in dyslexia and plays a strong role in single-word reading ability, which is the core diagnostic criterion for dyslexia. To our knowledge, only one previous study has examined specific relationships between reading-related skills and MMN measures at kindergarten age, a time when assessing risk for dyslexia is of great interest. This study of Finnish children aged 5–6, with 26 typically developing and 11 at-risk children, used tone stimuli with three types of deviants: intensity, frequency, and duration (Hämäläinen et al., 2015). Greater late MMN amplitude to the frequency deviants was significantly correlated with faster rapid object naming. No correlations were observed between any MMN measures and a test of phonological identification, nor were there any relations between RAN and the intensity or duration deviants.

### Mismatch Negativity Laterality in Relation to Reading, PA, and RAN

Few studies have directly investigated the relation between MMN laterality and reading-related abilities of PA and RAN. In adults with dyslexia, right lateralization of the magnetic MMN signal was significantly related with reading speed and accuracy (Thiede et al., 2020). One study of German-speaking children in grades 3–4 (age 9) found reduced right-lateralization of the MMN for /ba/ and /da/ syllables among a group of children with dyslexia who were defined primarily on phonological reading and spelling measures (Paul et al., 2006). In another line of work with German-speaking children who had a family history of (and thus risk for) dyslexia, the MMN to /ba/ and /da/ speech syllables was also examined (Maurer et al., 2003), the overall distribution of the early MMN response on the scalp was quite different than was seen here (despite the same average scalp reference), with the typical and at-risk groups showing large *positive* responses over a broad fronto-central area similar to where we observed negativity here. This group also measured the centroid of the MMN across the posterior regions of the head and found that leftward laterality of the MMN was related to better subsequent reading ability (Maurer et al., 2009). Because the frontal regions where the MMN is typically the strongest were not examined, it is difficult to directly compare with the current study.

Another study (Lachmann et al., 2005) compared groups of 9-year-old German-speaking children (age 9) defined by typical reading (*n* = 12); slow non-word reading, thought to relate to phonological deficits (*n* = 8); or slow sight word reading, thought to relate to visual/orthographic deficits (*n* = 8). The MMN to speech syllables in early time windows was measured, but the windows were selected separately for each group. The control group and the slow non-word reading group had MMN amplitudes that were significantly different from zero in right but left frontal electrodes. The slow sight-word reading group did not have significant MMNs at any site. Thus, these results are somewhat consistent with our findings in that the controls exhibited rightward lateralization and the deficit groups had reduced amplitudes, but the small groups and the fact that the late MMN was not examined prevent more direct comparisons. The slower reader group in that study may be somewhat analogous to Low RAN in this study. To our knowledge, no previous studies have specifically examined how laterality relates to RAN.

### Potential Impact of Methods on MMN Findings

There are a number of reasons that may explain why there were significant correlations between MMN measures and both PA and RAN in our study that were not seen in previous work. The stimuli and parameters for data acquisition and analysis used in different studies (including type of stimulus, stimulus onset asynchrony, reference electrodes, etc.) can greatly impact the timing and characteristics of the MMN (Bishop, 2007), and presumably the correlations between the MMN and behavior. The participant sample used in this study was larger than those of most previous studies, so other studies may have lacked power to detect significant associations. Previous studies also often used tone stimuli; importantly, direct comparison between tone- and syllable-evoked MMNs finds that the response to syllable stimuli is more closely related to reading ability in older children and adults (Schulte-Körne et al., 1998; Lachmann et al., 2005). Further, a meta-analysis that found that only MMN responses to speech stimuli were significantly related to dyslexia (Gu and Bi, 2020). Differences here may be related to the fact that reading English, a relatively opaque orthography, requires different reading skills than does Finnish or German, more transparent orthographies in which the MMN has been studied in more detail.

### Potential Explanations for Why MMN Relates to PA and RAN

Some insights into why PA might relate to the MMN come from structural and functional MRI studies. The MMN to speech depends on the individual’s ability to discriminate the standard and deviant stimuli, but it has also been suggested to reflect the quality of an individual’s phonological representations (Pakarinen et al., 2007). Debates are ongoing in the literature as to whether PA deficits in dyslexia relate to difficulty with perceiving, storing, and/or accessing phonological information (e.g., Joanisse et al., 2000; Ramus and Szenkovits, 2008). One influential brain-imaging study suggested that adults with dyslexia have intact, accurate phonological representations, but experience difficulty accessing these representations reliably due to reduced structural and functional connectivity between auditory/temporal cortex and inferior frontal regions that support higher-level phonological analysis (Boets et al., 2013). Additional studies have shown that the left arcuate fasciculus, which connects temporal and frontal regions, has reduced volume and organization in pre-reading children with poor PA skills (Vandermosten et al., 2012; Saygin et al., 2013; Wang et al., 2017), in school-age children with poor PA skills and genetic risk factors associated with dyslexia (Skeide et al., 2015) and in adults with dyslexia (Yeatman et al., 2011). In paradigms such as ours, the MMN may reflect the brain’s ability to quickly and accurately access phonological representations for higher-level processing.

Similarly, fMRI studies shed some light on to why the MMN might relate to RAN. RAN is thought to reflect the automaticity of cognitive and neural processes that support reading (Norton and Wolf, 2012). Brain activation patterns during RAN and word reading are strongly correlated in regions that support lower-level, highly automatized processes and timing (cerebellum, motor regions) as well as regions that support semantic and symbolic retrieval (middle temporal gyrus and supramarginal gyrus) (Cummine et al., 2015). Frontal-cerebellar functional connectivity during reading is also related to RAN ability in children (Norton et al., 2014). RAN may relate to the early MMN amplitude because RAN reflects the efficiency of neural systems that support automatic, pre-attentive processing of stimuli. When those early, upstream processes are highly efficient, more time and cognitive resources may be available for higher-level, downstream processing. A similar mechanism has been suggested for the behavioral basis of reading fluency, such that greater automaticity of sub-word and word-level skills leaves more time and cognitive resources for higher-level comprehension processes (Wolf et al., 2009). EEG theta power, which is associated with speech processing at the level of the phoneme and syllable, has been observed to be significantly right-lateralized in adult typical readers, and was less lateralized in dyslexia (Lehongre et al., 2013). The role of right frontal asymmetry in relation to stronger RAN skills should be further investigated.

### Limitations

The present study is limited by the fact that children were not explicitly tested on their ability to discriminate between the stimuli of interest. The natural speech syllables/ba/and/da/should be quite easy to discriminate, but documenting how subtle individual differences in auditory perceptual ability affect the MMN may help to further clarify its neural bases. Another potential limitation to consider of the correlational analyses is that the sample included a higher proportion of at-risk children. Finally, in this sample, PA was significantly associated with non-verbal IQ and early reading ability, but not with RAN, though the partial correlation analyses still showed associations with the late MMN mean amplitude measure.

## Conclusion

In sum, our results suggest that PA and RAN each have a unique and important relation to the MMN, as they do to reading. The MMN may provide an index of PA and RAN skills in a single, relatively unbiased measurement, reflecting both the accuracy and the automaticity of auditory (here, phonological) change detection. Crucially, the MMN can be measured earlier than either PA or RAN, and thus may be a very early indicator of reading-related abilities. Future work should continue to evaluate the viability of the MMN as an early predictor of reading difficulties across various languages. This will require studying large, diverse samples of children over time. However, if the psychometric properties of the MMN can be validated, the potential benefit of being able to identify children at risk for reading or language difficulties using a relatively fast and inexpensive brain measure that can be administered earlier than behavioral measures such as RAN and PA could allow earlier, more effective intervention for the many children who would otherwise struggle with reading difficulties.

## Data Availability Statement

The datasets presented in this article will be available upon request once other analyses related to the study are completed. Requests to access the datasets should be directed to EN, enorton@northwestern.edu.

## Ethics Statement

The studies involving human participants were reviewed and approved by the Committee on the Use of Human Experimental Subjects at MIT and the Institutional Review Board at Boston Children’s Hospital. Written informed consent to participate in this study was provided by the participants’ legal guardian/parent.

## Author Contributions

EN, NG, and JG designed the larger research study. ME designed the ERP MMN paradigm. EN, SB, OO-P, and ME collected the data. EN, SB, ME, and SM conducted the preprocessing. EN performed the analyses, and drafted the manuscript with input from NG and JG. All authors reviewed and approved the manuscript.

## Conflict of Interest

The authors declare that the research was conducted in the absence of any commercial or financial relationships that could be construed as a potential conflict of interest.

## Footnotes

1 http://sccn.ucsd.edu/eeglab

2 http://erpinfo.org/erplab

## References

[B1] Alonso-Bua B. Diaz F. Ferraces M. (2006). The contribution of AERPs (MMN and LDN) to studying temporal vs. linguistic processing deficits in children with reading difficulties. *Int. J. Psychophysiol.* 59 159–167. 10.1016/j.ijpsycho.2005.03.020 16112215

[B2] Bishop D. V. Hardiman M. J. Barry J. G. (2011). Is auditory discrimination mature by middle childhood? A study using time-frequency analysis of mismatch responses from 7 years to adulthood. *Dev. Sci.* 14 402–416. 10.1111/j.1467-7687.2010.00990.x 22213909 PMC3083517

[B3] Bishop D. V. M. (2007). Using mismatch negativity to study central auditory processing in developmental language and literacy impairments: Where are we, and where should we be going? *Psychol. Bull.* 133 651–672. 10.1037/0033-2909.133.4.651 17592960

[B4] Bitz U. Gust K. Spitzer M. Kiefer M. (2007). Phonological deficit in school children is reflected in the mismatch negativity. *Neuro. Rep.* 18 911–915. 10.1097/WNR.0b013e32810f2e25 17515800

[B5] Boersma P. Weenink D. (2010). *Praat: Doing Phonetics by Computer: Praat Software Version 5.* Available online at: http://www.fon.hum.uva.nl/praat/

[B6] Boets B. de Beeck H. P. O. Vandermosten M. Scott S. K. Gillebert C. R. Mantini D. (2013). Intact but less accessible phonetic representations in adults with dyslexia. *Science* 342 1251–1254. 10.1126/science.1244333 24311693 PMC3932003

[B7] Bradley L. Bryant P. E. (1978). Difficulties in auditory organisation as a possible cause of reading backwardness. *Nature* 271 746–747. 625341 10.1038/271746a0

[B8] Bruder J. Leppänen P. H. Bartling J. Csepe V. Demonet J. F. Schulte-Korne G. (2011). Children with dyslexia reveal abnormal native language representations: evidence from a study of mismatch negativity. *Psychophysiology* 48 1107–1118. 10.1111/j.1469-8986.2011.01179.x 21332488

[B9] Catts H. W. McIlraith A. Bridges M. S. Nielsen D. C. (2017). Viewing a phonological deficit within a multifactorial model of dyslexia. *Read. Write* 30 613–629. 10.1007/s11145-016-9692-2

[B10] Cheour M. Èeponienë R. Hukki J. Haapanen M.-L. Näätänen R. Alho K. (1999). Brain dysfunction in neonates with cleft palate revealed by the mismatch negativity. *Clin. Neurophysiol.* 110 324–328. 10.1016/S1388-2457(98)00005-4 10210622

[B11] Cheour M. Korpilahti P. Martynova O. Lang A. H. (2001). Mismatch negativity and late discriminative negativity in investigating speech perception and learning in children and infants. *Audiol. Neurotol.* 6 2–11. 10.1159/000046804 11173771

[B12] Cohen J. E. (1988). *Statistical power analysis for the behavioral sciences.* Mahwah, NJ: Lawrence Erlbaum Associates.

[B13] Compton D. L. (2020). Focusing our view of dyslexia through a multifactorial lens: a commentary. *Learn. Disabil. Q.* 2020:073194872093900. 10.1177/0731948720939009

[B14] Cummine J. Chouinard B. Szepesvari E. Georgiou G. K. (2015). An examination of the rapid naming-reading relationship using functional magnetic resonance imaging. *Neuroscience* 305 49–66. 10.1016/j.neuroscience.2015.07.071 26235433

[B15] Cuthbert B. N. Insel T. R. (2013). Toward the future of psychiatric diagnosis: the seven pillars of RDoC. *BMC Med.* 11:126. 10.1186/1741-7015-11-126 23672542 PMC3653747

[B16] Damme K. S. F. Norton E. S. Briggs-Gowan M. J. Wakschlag L. S. Mittal V. A. (2020). Developmental patterning of irritability enhances prediction of psychopathology in pre-adolescence: Improving RDoC with developmental science. *J. Abnormal Psychol.* 10.1101/2020.04.30.070714v1 PMC9439570 35901387

[B17] Delorme A. Makeig S. (2004). EEGLAB: an open source toolbox for analysis of single-trial EEG dynamics. *J. Neurosci. Methods* 134 9–21. 10.1016/j.jneumeth.2003.10.009 15102499

[B18] Duncan L. G. Seymour P. H. (2000). Socio-economic differences in foundation-level literacy. *Br. J. Psychol.* 91 145–166. 10832511 10.1348/000712600161736

[B19] Giraud A. L. Ramus F. (2013). Neurogenetics and auditory processing in developmental dyslexia. *Curr. Opin. Neurobiol.* 23 37–42. 10.1016/j.conb.2012.09.003 23040541

[B20] Goswami U. (2015). Sensory theories of developmental dyslexia: three challenges for research. *Nat. Rev. Neurosci.* 16 43–54. 25370786 10.1038/nrn3836

[B21] Gu C. Bi H. Y. (2020). Auditory processing deficit in individuals with dyslexia: a meta-analysis of mismatch negativity. *Neurosci. Biobehav. Rev.* 116 396–405. 10.1016/j.neubiorev.2020.06.032 32610180

[B22] Guttorm T. K. Leppänen P. H. Hämäläinen J. A. Eklund K. M. Lyytinen H. J. (2010). Newborn event-related potentials predict poorer pre-reading skills in children at risk for dyslexia. *J. Learn. Disabil.* 43 391–401. 10.1177/0022219409345005 19890075

[B23] Guttorm T. K. Leppänen P. H. Poikkeus A. M. Eklund K. M. Lyytinen P. Lyytinen H. (2005). Brain event-related potentials (ERPs) measured at birth predict later language development in children with and without familial risk for dyslexia. *Cortex* 41 291–303. 10.1016/S0010-9452(08)70267-3 15871595

[B24] Halliday L. F. Barry J. G. Hardiman M. J. Bishop D. V. (2014). Late, not early mismatch responses to changes in frequency are reduced or deviant in children with dyslexia: an event-related potential study. *J. Neurodev. Disord.* 6:21. 10.1186/1866-1955-6-21 25110526 PMC4126817

[B25] Hämäläinen J. A. Guttorm T. K. Richardson U. Alku P. Lyytinen H. Leppänen P. H. T. (2013). Auditory event-related potentials measured in kindergarten predict later reading problems at school age. *Dev. Neuropsychol.* 38 550–566. 10.1080/87565641.2012.718817 24219695

[B26] Hämäläinen J. A. Lohvansuu K. Ervast L. Leppänen P. H. (2015). Event-related potentials to tones show differences between children with multiple risk factors for dyslexia and control children before the onset of formal reading instruction. *Int. J. Psychophysiol.* 95 101–112. 10.1016/j.ijpsycho.2014.04.004 24746550

[B27] Hancock R. Pugh K. R. Hoeft F. (2017). Neural noise hypothesis of developmental dyslexia. *Trends Cogn. Sci.* 21 434–448. 10.1016/j.tics.2017.03.008 28400089 PMC5489551

[B28] Hommet C. Vidal J. Roux S. Blanc R. Barthez M. A. De Becque B. (2009). Topography of syllable change-detection electrophysiological indices in children and adults with reading disabilities. *Neuropsychologia* 47 761–770. 10.1016/j.neuropsychologia.2008.12.010 19126410

[B29] Huttunen-Scott T. Kaartinen J. Tolvanen A. Lyytinen H. (2008). Mismatch negativity (MMN) elicited by duration deviations in children with reading disorder, attention deficit or both. *Int. J. Psychophysiol.* 69 69–77. 10.1016/j.ijpsycho.2008.03.002 18440662

[B30] Ikezawa S. Nakagome K. Mimuraa M. Shinoda J. Itoh K. Homma I. (2008). Gender differences in lateralization of mismatch negativity in dichotic listening tasks. *Int. J. Psychophysiol.* 68 41–50. 10.1016/j.ijpsycho.2008.01.006 18295364

[B31] Jääskeläinen I. P. Ahveninen J. Bonmassar G. Dale A. M. Ilmoniemi R. J. Levänen S. (2004). Human posterior auditory cortex gates novel sounds to consciousness. *Proc. Natl. Acad. Sci.* 101 6809–6814. 15096618 10.1073/pnas.0303760101 PMC404127

[B32] Jemel B. Achenbach C. Müller B. W. Röpcke B. Oades R. D. (2002). Mismatch negativity results from bilateral asymmetric dipole sources in the frontal and temporal lobes. *Brain Topogr.* 15 13–27. 12371672 10.1023/a:1019944805499

[B33] Joanisse M. F. Manis F. R. Keating P. Seidenberg M. S. (2000). Language deficits in dyslexic children: Speech perception, phonology, and morphology. *J. Exper. Child Psychol.* 77 30–60. 10.1006/jecp.1999.2553 10964458

[B34] Kappenman E. S. Luck S. J. (2010). The effects of electrode impedance on data quality and statistical significance in ERP recordings. *Psychophysiology* 47 888–904. 10.1111/j.1469-8986.2010.01009.x 20374541 PMC2902592

[B35] Kappenman E. S. Luck S. J. (2016). Best practices for event-related potential research in clinical populations. *Biol. Psychiatry Cogn. Neurosci. Neuroimag.* 1 110–115. 10.1016/j.bpsc.2015.11.007 27004261 PMC4797328

[B36] Kaufman A. S. Kaufman N. L. (2004). *Kaufman brief intelligence test*, 2nd Edn. Pines, MN: American Guidance Service.

[B37] Keil A. Debener S. Gratton G. Junghöfer M. Kappenman E. S. Luck S. J. (2014). Committee report: Publication guidelines and recommendations for studies using electroencephalography and magnetoencephalography. *Psychophysiology* 51 1–21. 24147581 10.1111/psyp.12147

[B38] Korpilahti P. Krause C. M. Holopainen I. Lang A. H. (2001). Early and late mismatch negativity elicited by words and speech-like stimuli in children. *Brain Lang.* 76 332–339. 11247648 10.1006/brln.2000.2426

[B39] Korpilahti P. Lang H. Aaltonen O. (1995). Is there a late-latency mismatch negativity (MMN) component? *Electroencephalogr. Clin. Neurophysiol.* 95:96. 10.1016/0013-4694(95)90016-G 7523075

[B40] Kraus N. McGee T. Carrell T. Zecker S. Nicol T. G. Koch D. B. (1996). Auditory neurophysiologic responses and discrimination deficits in children with learning problems. *Science* 273 971–973. 10.1126/science.273.5277.971 8688085

[B41] Kujala T. Näätänen R. (2001). The mismatch negativity in evaluating central auditory dysfunctions in dyslexia. *Neurosci. Behav. Rev.* 25 535–543. 10.1016/S0149-7634(01)00032-X 11595273

[B42] Lachmann T. Berti S. Kujala T. Schroger E. (2005). Diagnostic subgroups of developmental dyslexia have different deficits in neural processing of tones and phonemes. *Int. J. Psychophysiol.* 56 105–120. 10.1016/j.ijpsycho.2004.11.005 15804446

[B43] Landerl K. Ramus F. Moll K. Lyytinen H. Leppänen P. H. Lohvansuu K. (2013). Predictors of developmental dyslexia in European orthographies with varying complexity. *J. Child Psychol. Psychiatry Allied Discip.* 54 686–694. 10.1111/jcpp.12029 23227813

[B44] Lehongre K. Morillon B. Giraud A.-L. Ramus F. (2013). Impaired auditory sampling in dyslexia: further evidence from combined fMRI and EEG. *Front. Hum. Neurosci.* 7:454. 10.3389/fnhum.2013.00454 23950742 PMC3738857

[B45] Lehongre K. Ramus F. Villiermet N. Schwartz D. Giraud A. L. (2011). Altered low-gamma sampling in auditory cortex accounts for the three main facets of dyslexia. *Neuron* 72 1080–1090. 10.1016/j.neuron.2011.11.002 22196341

[B46] Leppänen P. H. Hämäläinen J. A. Guttorm T. K. Eklund K. M. Salminen H. Tanskanen A. (2012). Infant brain responses associated with reading-related skills before school and at school age. *Clin. Neurophysiol.* 42 35–41. 10.1016/j.neucli.2011.08.005 22200340

[B47] Leppänen P. H. Pihko E. Eklund K. M. Lyytinen H. (1999). Cortical responses of infants with and without a genetic risk for dyslexia: II. Group effects. *Neuro. Rep.* 10 969–973. 10.1097/00001756-199904060-00014 10321469

[B48] Leppänen P. H. Richardson U. Pihko E. Eklund K. M. Guttorm T. K. Aro M. (2002). Brain responses to changes in speech sound durations differ between infants with and without familial risk for dyslexia. *Dev. Neuropsychol.* 22 407–422. 10.1207/S15326942dn2201_4 12405511

[B49] Leppänen P. H. T. Hämäläinen J. A. Salminen H. K. Eklund K. M. Guttorm T. K. Lohvansuu K. (2010). Newborn brain event-related potentials revealing atypical processing of sound frequency and the subsequent association with later literacy skills in children with familial dyslexia. *Cortex* 46 1362–1376. 10.1016/j.cortex.2010.06.003 20656284

[B50] Lopez-Calderon J. Luck S. J. (2014). ERPLAB: An open-source toolbox for the analysis of event-related potentials. *Front. Hum. Neurosci.* 8:213. 10.3389/fnhum.2014.00213 24782741 PMC3995046

[B51] Luck S. J. (2014). *An introduction to the event-related potential technique*, 2nd Edn. Cambridge: MIT Press.

[B52] Luck S. J. Gaspelin N. (2017). How to get statistically significant effects in any ERP experiment (and why you shouldn’t). *Psychophysiology* 54 146–157. 10.1111/psyp.12639 28000253 PMC5178877

[B53] Manis F. R. Doi L. M. Bhadha B. (2000). Naming speed, phonological awareness, and orthographic knowledge in second graders. *J. Learn. Disabil.* 33 325–333. 10.1177/002221940003300405 15493095

[B54] Maurer U. Bucher K. Brem S. Benz R. Kranz F. Schulz E. (2009). Neurophysiology in preschool improves behavioral prediction of reading ability throughout primary school. *Biol. Psychiatry* 66 341–348. 10.1016/j.biopsych.2009.02.031 19423082

[B55] Maurer U. Bucher K. Brem S. Brandeis D. (2003). Altered responses to tone and phoneme mismatch in kindergartners at familial dyslexia risk. *Neuro Rep.* 14 2245–2250. 10.1097/00001756-200312020-00022 14625456

[B56] McWeeny S. Norton E. S. (2020). Understanding event related potentials (ERPs) in clinical and basic and language and communication disorders research: a tutorial. *Int. J. Lang. Commun. Disord.* 55 445–457. 10.1111/1460-6984.12535 32347637 PMC7802513

[B57] Meyer L. Schaadt G. (2020). Aberrant prestimulus oscillations in developmental dyslexia support an underlying attention shifting deficit. *Cerebral Cortex Commun.* 1:tgaa006. 10.1093/texcom/tgaa006 PMC8152944 34296087

[B58] Mittal V. A. Wakschlag L. S. (2017). Research domain criteria (RDoC) grows up: Strengthening neurodevelopment investigation within the RDoC framework. *J. Affective Disord.* 216 30–35. 10.1016/j.jad.2016.12.011 28010957 PMC5471127

[B59] Morr M. L. Shafer V. L. Kreuzer J. A. Kurtzberg D. (2002). Maturation of mismatch negativity in typically developing infants and preschool children. *Ear Hear.* 23 118–136. 10.1097/00003446-200204000-00005 11951848

[B60] Morris R. D. Stuebing K. K. Fletcher J. M. Shaywitz S. E. Lyon G. R. Shankweiler D. P. (1998). Subtypes of reading disability: variability around a phonological core. *J. Educ. Psychol.* 90 347–373. 10.1037/0022-0663.90.3.347

[B61] Näätänen R. Gaillard A. W. K. Mäntysalo S. (1978). Early selective-attention effect on evoked potential reinterpreted. *Acta Psychol.* 42 313–329. 10.1016/0001-6918(78)90006-9 685709

[B62] Näätänen R. Kreegipuu K. (2011). “The mismatch negativity (MMN),” in *The Oxford handbook of event-related potential components*, eds Luck S. J. Kappenman E. S. (Oxford: Oxford University Press), 143–158.

[B63] Näätänen R. Kujala T. Escera C. Baldeweg T. Kreegipuu K. Carlson S. (2012). The mismatch negativity (MMN), A unique window to disturbed central auditory processing in ageing and different clinical conditions. *Clin. Neurophysiol.* 123 424–458. 10.1016/j.clinph.2011.09.020 22169062

[B64] Näätänen R. Paavilainen P. Rinne T. Alho K. (2007). The mismatch negativity (MMN) in basic research of central auditory processing: a review. *Clin. Neurophysiol.* 118 2544–2590. 17931964 10.1016/j.clinph.2007.04.026

[B65] Naples A. J. Chang J. T. Katz L. Grigorenko E. L. (2009). Same or different? Insights into the etiology of phonological awareness and rapid naming. *Biol. Psychol.* 80 226–239. 10.1016/j.biopsycho.2008.10.002 19007845 PMC2708917

[B66] Neuhoff N. Bruder J. Bartling J. Warnke A. Remschmidt H. Müller-Myhsok B. (2012). Evidence for the late MMN as a neurophysiological endophenotype for dyslexia. *PLoS One* 7:e34909. 10.1371/journal.pone.0034909 22606227 PMC3351484

[B67] Noordenbos M. W. Segers E. Serniclaes W. Mitterer H. Verhoeven L. (2012). Neural evidence of allophonic perception in children at risk for dyslexia. *Neuropsychologia* 50 2010–2017. 22569214 10.1016/j.neuropsychologia.2012.04.026

[B68] Norton E. S. Black J. M. Stanley L. M. Tanaka H. Gabrieli J. D. E. Sawyer C. (2014). Functional neuroanatomical evidence for the double-deficit hypothesis of developmental dyslexia. *Neuropsychologia* 61 235–246. 10.1016/j.neuropsychologia.2014.06.015 24953957 PMC4339699

[B69] Norton E. S. Wolf M. (2012). Rapid automatized naming (RAN) and reading fluency: Implications for understanding and treatment of reading disabilities. *Ann. Rev. Psychol.* 63 427–452. 10.1146/annurev-psych-120710-100431 21838545

[B70] O’Brien G. Yeatman J. D. (2020). Bridging sensory and language theories of dyslexia: toward a multifactorial model. *Dev. Sci.* 2020:e13039. 10.1111/desc.13039 33021019 PMC8244000

[B71] Opitz B. Rinne T. Mecklinger A. von Cramon D. Y. Schröger E. (2002). Differential contribution of frontal and temporal cortices to auditory change detection: fMRI and ERP results. *NeuroImage* 15 167–174. 10.1006/NIMG.2001.0970 11771985

[B72] Ozernov-Palchik O. Norton E. S. Sideridis G. Beach S. D. Wolf M. Gabrieli J. D. E. (2017). Longitudinal stability of pre-reading skill profiles of kindergarten children: implications for early screening and theories of reading. *Dev. Sci.* 20:e12471. 10.1111/desc.12471 PMC5393968 27747988

[B73] Pakarinen S. Takegata R. Rinne T. Huotilainen M. Näätänen R. (2007). Measurement of extensive auditory discrimination profiles using the mismatch negativity (MMN) of the auditory event-related potential (ERP). *Clin. Neurophysiol.* 118 177–185. 10.1016/j.clinph.2006.09.001 17070103

[B74] Paris S. G. (2005). Reinterpreting the development of reading skills. *Reading Res. Q.* 40 184–202.

[B75] Paul I. Bott C. Heim S. Wienbruch C. Elbert T. (2006). Phonological but not auditory discrimination is impaired in dyslexia. *Eur. J. Neurosci.* 24 2945–2953. 17116164 10.1111/j.1460-9568.2006.05153.x

[B76] Pennington B. F. Santerre-Lemmon L. Rosenberg J. MacDonald B. Boada R. Friend A. (2012). Individual prediction of dyslexia by single versus multiple deficit models. *J. Abnormal Psychol.* 121 212–224. 10.1037/a0025823 22022952 PMC3270218

[B77] Petrill S. A. Deater-Deckard K. Thompson L. A. DeThorne L. S. Schatschneider C. (2006). Genetic and environmental effects of serial naming and phonological awareness on early reading outcomes. *J. Educ. Psychol.* 98:112. 10.1037/0022-0663.98.1.112 19444324 PMC2681098

[B78] Plakas A. van Zuijen T. van Leeuwen T. Thomson J. M. van der Leij A. (2013). Impaired non-speech auditory processing at a pre-reading age is a risk-factor for dyslexia but not a predictor: an ERP study. *Cortex* 49 1034–1045. 10.1016/j.cortex.2012.02.013 22542727

[B79] Ramus F. Szenkovits G. (2008). What phonological deficit? *Q. J. Exper. Psychol.* 61 129–141. 10.1080/17470210701508822 18038344

[B80] Rinne T. Alho K. Ilmoniemi R. J. Virtanen J. Näätänen R. (2000). Separate time behaviors of the temporal and frontal mismatch negativity sources. *Neuroimage* 12 14–19. 10.1006/nimg.2000.0591 10875898

[B81] Robins S. Ghosh D. Rosales N. Treiman R. (2014). Letter knowledge in parent–child conversations: differences between families differing in socio-economic status. *Front. Psychol.* 5:632. 10.3389/fpsyg.2014.00632 25009516 PMC4067604

[B82] Saygin Z. M. Norton E. S. Osher D. E. Beach S. D. Cyr A. B. Ozernov-Palchik O. (2013). Tracking the roots of reading ability: White matter volume and integrity correlate with phonological awareness in prereading and early-reading kindergarten children. *J. Neurosci.* 33 13251–13258. 10.1523/JNEUROSCI.4383-12.2013 23946384 PMC3742917

[B83] Schaadt G. Männel C. (2019). Phonemes, words, and phrases: tracking phonological processing in pre-schoolers developing dyslexia. *Clin. Neurophysiol.* 130 1329–1341. 31200240 10.1016/j.clinph.2019.05.018

[B84] Schaadt G. Männel C. van der Meer E. Pannekamp A. Oberecker R. Friederici A. D. (2015). Present and past: Can writing abilities in school children be associated with their auditory discrimination capacities in infancy? *Res. Dev. Disabil.* 47 318–333. 26479824 10.1016/j.ridd.2015.10.002

[B85] Schatschneider C. Fletcher J. M. Francis D. J. Carlson C. D. Foorman B. R. (2004). Kindergarten prediction of reading skills: A longitudinal comparative analysis. *J. Educ. Psychol.* 96 265–282. 10.1037/0022-0663.96.2.265

[B86] Schulte-Körne G. Deimel W. Bartling J. Remschmidt H. (1998). Auditory processing and dyslexia: evidence for a specific speech processing deficit. *Neuro. Rep.* 9 337–340. 10.1097/00001756-199801260-00029 9507979

[B87] Schulte-Körne G. Deimel W. Bartling J. Remschmidt H. (1999). Pre-attentive processing of auditory patterns in dyslexic human subjects. *Neurosci. Lett.* 276 41–44. 10.1016/S0304-3940(99)00785-5 10586970

[B88] Schulte-Körne G. Deimel W. Bartling J. Remschmidt H. (2001). Speech perception deficit in dyslexic adults as measured by mismatch negativity (MMN). *Int. J. Psychophysiol.* 40 77–87. 10.1016/S0167-8760(00)00152-5 11166109

[B89] Schwartz S. Shinn-Cunningham B. Tager-Flusberg H. (2018). Meta-analysis and systematic review of the literature characterizing auditory mismatch negativity in individuals with autism. *Neurosci. Biobehav. Rev.* 87 106–117. 29408312 10.1016/j.neubiorev.2018.01.008 PMC5845770

[B90] Sharma M. Purdy S. C. Newall P. Wheldall K. Beaman R. Dillon H. (2006). Electrophysiological and behavioral evidence of auditory processing deficits in children with reading disorder. *Clin. Neurophysiol.* 117 1130–1144. 10.1016/j.clinph.2006.02.001 16564738

[B91] Skeide M. A. Kirsten H. Kraft I. Schaadt G. Müller B. Neef N. (2015). Genetic dyslexia risk variant is related to neural connectivity patterns underlying phonological awareness in children. *Neuroimage* 118 414–421. 26080313 10.1016/j.neuroimage.2015.06.024

[B92] Stoodley C. J. Hill P. R. Stein J. F. Bishop D. V. (2006). Auditory event-related potentials differ in dyslexics even when auditory psychophysical performance is normal. *Brain Res.* 1121 190–199. 10.1016/j.brainres.2006.08.095 17010945

[B93] Thiede A. Parkkonen L. Virtala P. Laasonen M. Mäkelä J. P. Kujala T. (2020). Neuromagnetic speech discrimination responses are associated with reading-related skills in dyslexic and typical readers. *Heliyon* 6:e04619. 10.1016/j.heliyon.2020.e04619 32904386 PMC7452546

[B94] Todd J. Fitzgerald K. (2020). Making sense of mismatch negativity. *Front. Psychiatry* 11:468. 10.3389/fpsyt.2020.00468 32595529 PMC7300203

[B95] van Bergen E. van der Leij A. de Jong P. F. (2014). The intergenerational multiple deficit model and the case of dyslexia. *Front. Hum. Neurosci.* 8:346. 10.3389/fnhum.2014.00346 24920944 PMC4041008

[B96] van Leeuwen T. Been P. van Herten M. Zwarts F. Maassen B. van der Leij A. (2008). Two-month-old infants at risk for dyslexia do not discriminate /bAk/ from /dAk/: A brain-mapping study. *J. Neuroling.* 21 333–348. 10.1016/j.jneuroling.2007.07.004

[B97] van Zuijen T. L. Plakas A. Maassen B. A. M. Maurits N. M. van der Leij A. (2013). Infant ERPs separate children at risk of dyslexia who become good readers from those who become poor readers. *Dev. Sci.* 16 554–563. 10.1111/desc.12049 23786473

[B98] Vandermosten M. Boets B. Poelmans H. Sunaert S. Wouters J. Ghesquière P. (2012). A tractography study in dyslexia: neuroanatomic correlates of orthographic, phonological and speech processing. *Brain* 135 935–948. 10.1093/brain/awr363 22327793

[B99] Volkmer S. Schulte-Körne G. (2018). Cortical responses to tone and phoneme mismatch as a predictor of dyslexia? A systematic review. *Schizophrenia Res.* 191 148–160. 10.1016/j.schres.2017.07.010 28712970

[B100] Wagner R. Torgesen J. Rashotte C. A. (1999). *Comprehensive Test of Phonological Processing.* Austin, TX: Pro-Ed.

[B101] Wang Y. Mauer M. V. Raney T. Peysakhovich B. Becker B. L. C. Sliva D. D. (2017). Development of tract-specific white matter pathways during early reading development in at-risk children and typical controls. *Cerebral Cortex* 27 2469–2485. 10.1093/cercor/bhw095 27114172 PMC5964366

[B102] Wanzek J. Vaughn S. (2007). Research-based implications from extensive early reading interventions. *School Psychol. J.* 36 541–561. 10.1080/02796015.2007.12087917

[B103] Wolf M. Barzillai M. Gottwald S. Miller L. Spencer K. Norton E. (2009). The RAVE-O intervention: Connecting neuroscience to the classroom. *Mind Brain Educ.* 3 84–93. 10.1111/j.1751-228X.2009.01058.x PMC11456325 39372597

[B104] Wolf M. Bowers P. G. (1999). The double-deficit hypothesis for the developmental dyslexias. *J. Educ. Psychol.* 91 415–438. 10.1037/0022-0663.91.3.415

[B105] Wolf M. Denckla M. B. (2005). *RAN/RAS: Rapid Automatized Naming and Rapid Alternating Stimulus Tests.* Austin, TX: Pro.Ed.

[B106] Woodcock R. W. (1998). *Woodcock Reading Mastery Tests – Revised/Normative Update.* Circle Pines, MN: American Guidance Service.

[B107] Yeatman J. D. Dougherty R. F. Rykhlevskaia E. Sherbondy A. J. Deutsch G. K. Wandell B. A. (2011). Anatomical properties of the arcuate fasciculus predict phonological and reading skills in children. *J. Cogn. Neurosci.* 23 3304–3317. 21568636 10.1162/jocn_a_00061 PMC3214008

